# Single-Layer Magnet Phase in Intrinsic Magnetic Topological Insulators, [MnTe][Bi_2_Te_3_]_*n*_, Far beyond the Thermodynamic Limit

**DOI:** 10.1021/acs.nanolett.4c05860

**Published:** 2025-03-13

**Authors:** Deepti Jain, Hee Taek Yi, Xiong Yao, Alessandro R. Mazza, An-Hsi Chen, Kim Kisslinger, Myung-Geun Han, Matthew Brahlek, Seongshik Oh

**Affiliations:** † Department of Physics and Astronomy, 242612 Rutgers, The State University of New Jersey, Piscataway, New Jersey 08854, United States; ‡ Materials Science and Technology Division, 6146 Oak Ridge National Laboratory, Oak Ridge, Tennessee 37831, United States; ∥ Center for Functional Nanomaterials, 8099 Brookhaven National Laboratory, Upton, New York 11973, United States; ⊥ Condensed Matter Physics and Materials Science, Brookhaven National Laboratory, Upton, New York 11973, United States; # Center for Quantum Materials Synthesis, Rutgers, The State University of New Jersey, Piscataway, New Jersey 08854, United States

**Keywords:** intrinsic magnetic topological insulators, molecular beam epitaxy, 2D ferromagnets, topological materials, topological insulator thin films

## Abstract

The intrinsic magnetic topological insulator (IMTI) family [MnTe]­[Bi_2_Te_3_]_*n*_ has demonstrated magneto-topological properties dependent on *n*, making it a promising platform for advanced electronics and spintronics. However, due to technical barriers in sample synthesis, their properties in the large *n* limit remain unknown. To overcome this, we utilized the atomic layer-by-layer molecular beam epitaxy (ALL-MBE) technique and achieved IMTIs with *n* as large as 15, far beyond that previously reported in bulk crystals or thin films. Then, we discover that the “single-layer magnet (SLM)” phase, primarily determined by intralayer ferromagnetic coupling, emerges for *n* > ∼4 and remains little affected up to *n* = 15. Nonetheless, still, nonzero, interlayer ferromagnetic coupling is necessary to stabilize the SLM phase, suggesting that the SLM phase eventually disappears in the *n* → ∞ limit. This study uncovers the secrets of IMTIs beyond the thermodynamic limit and opens a door to diverse magneto-topological applications.

## Graphical abstract

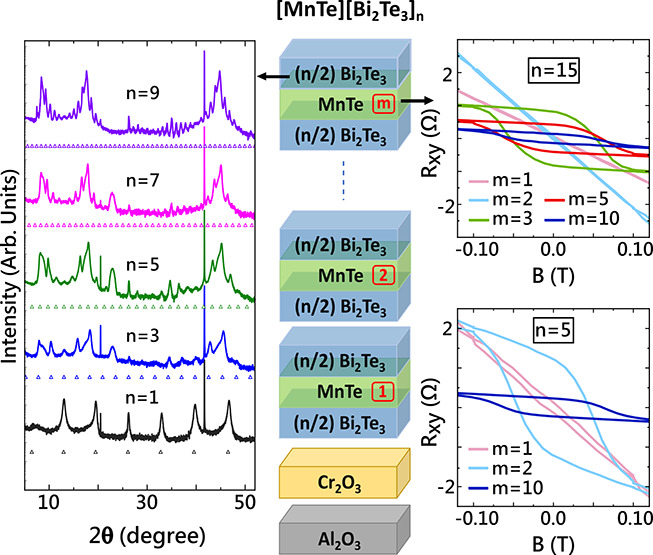

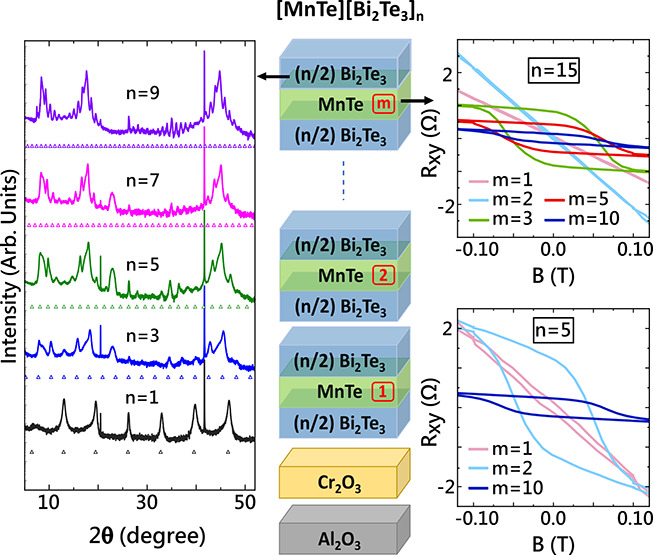

[MnTe]­[Bi_2_Te_3_]_*n*_, the only intrinsic magnetic topological insulators (IMTIs) known to exist so far, is a stoichiometric material system that is topologically nontrivial with an inherent magnetic order. These unique attributes make it an ideal platform to host a myriad of topological states such as axion insulators, magnetic Weyl semimetals, Chern insulators, topological magnetoelectric effect, and high temperature quantum anomalous Hall effects.^1–9^ Moreover, their built-in magnetism and naturally ordered structure ensures more homogeneous properties as compared to magnetically doped topological insulators, which is beneficial for reproducibility and scalability in practical applications.

Depending on *n*, i.e., the number of Bi_2_Te_3_ layers, the magnetic behavior of [MnTe]­[Bi_2_Te_3_]_*n*_ varies. In the first member of this family of materials, MnBi_2_Te_4_ (*n* = 1), each unit cell can be viewed as a layer of MnTe, an antiferromagnet, inserted within the topological insulator Bi_2_Te_3_. Together they stack as Te–Bi–Te–Mn–Te–Bi–Te, forming self-organized septuple layers (SLs) with each SL bonded to the next via weak van der Waals forces. This results in a magnetic structure akin to an A-type antiferromagnet: ferromagnetic (FM) order within each SL and antiferromagnetic (AFM) coupling between adjacent SLs.^6,10,11^ By inserting (*n* – 1) Bi_2_Te_3_ units between each SL, the other members of the [MnTe]­[Bi_2_Te_3_]_*n*_ family can be realized, as seen in the schematic of Figure 1a. As *n* increases, the AFM interlayer exchange coupling (IEC) between SLs is weakened, and the magnetic properties of [MnTe]­[Bi_2_Te_3_]_*n*_ can be tuned. Accordingly, the IEC progressively decreases in MnBi_4_Te_7_ (*n* = 2) and MnBi_6_Te_10_ (*n* = 3). Some studies report that *n* = 2 and *n* = 3 have a weakened AFM order (as compared to *n* = 1),^12–15^ while others note competing AFM and FM orders.^16,17^ Ultimately, the IEC completely vanishes for MnBi_8_Te_13_ (*n* = 4) resulting in an FM state.^12,18^ For *n* ≥ 4, once the individual FM SLs are decoupled, a “single-layer magnet (SLM)” phase has been predicted in which the SLs behave like independent 2D ferromagnets.^12,19^ In other words, it is expected that in the *n* → ∞ limit the FM ordering will persist. Unfortunately, this prediction of the SLM phase has not been verified beyond *n* = 5 due to challenges in synthesizing high order *n* members.

**Figure 1:**
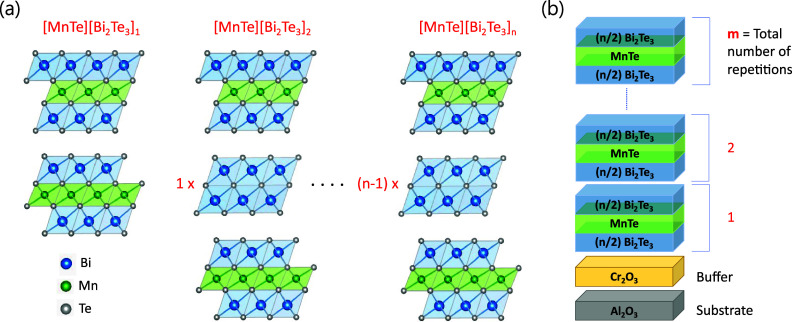
Crystal structure and growth schematic of [MnTe]­[Bi_2_Te_3_]_*n*_. (a) Illustration of the generalized structure for all [MnTe]­[Bi_2_Te_3_]_*n*_, with the number of Bi_2_Te_3_ QLs in between two SLs increasing with each order. (b) A schematic depicting the layer-by-layer growth of [MnTe]­[Bi_2_Te_3_]_*n*_ on Al_2_O_3_(0001) substrates. Each “block” contains one MnTe layer inserted between *n* Bi_2_Te_3_ QLs, which is then repeated *m* times depending on the required thickness or total number of SLs.

To date, the most extensively studied members of the [MnTe]­[Bi_2_Te_3_]_*n*_ material system are bulk crystals and exfoliated flakes of *n* = 1, 2, 3, and 4. This is because the temperature range in which [MnTe]­[Bi_2_Te_3_]_*n*_ compounds crystallize gets narrower as *n* increases. Hence, it becomes progressively more difficult to obtain high purity crystals for higher order *n* that are of adequate size.^20,21^ As a result, growth of only *n* ≤ 7 phases has been reported. Here, however, we demonstrate that these thermodynamic limitations can be bypassed with the atomic-layer-by-layer molecular beam epitaxy (ALL-MBE) technique and present previously unknown electronic and magnetic properties of these materials well beyond the bulk limit.

Figure 1b shows a schematic of the growth structure on Al_2_O_3_(0001) substrates. To improve sticking to the substrate, a buffer layer of ∼1 nm-thick Cr_2_O_3_ is introduced between the substrate and [MnTe]­[Bi_2_Te_3_]_*n*_ films, similar to our previous works (see complete details in the Experimental Methods section of the Supporting Information).^22–25^ Next, depending on the choice of *n*, each individual layer of MnTe and Bi_2_Te_3_ is deposited sequentially at 260–300 °C. This is achieved by opening and closing the elemental Mn and Bi shutters, while the Te shutter is kept open, with ∼6–7 times higher Te flux than Bi and Mn. This growth approach is based on the fact that the septuple layer, MnBi_2_Te_4_, is thermodynamically more stable than having a heterostructure of two separate layers of MnTe and Bi_2_Te_3_ on top of each other. Accordingly, when a MnTe layer is deposited between Bi_2_Te_3_ layers, it naturally forms a septuple layer of MnBi_2_Te_4_, while additional Bi_2_Te_3_ layers form quintuple layers below and above the MnBi_2_Te_4_ layer. This way, we are able to “pin” the Mn atoms and confine the SLs in the desired positions: this mode of growth is critical to achieving the high-order [MnTe]­[Bi_2_Te_3_]_*n*_ structures reported here. Another significant advantage of such a layer-by-layer approach compared to the more common codeposition method is that it is not critical to control the Mn:Bi ratio. As shown in the schematic of Figure 1b, the film is grown in “blocks”, each comprising a MnTe layer and *n* QLs of Bi_2_Te_3_. Each block is repeated *m* times to obtain the desired thickness. In the samples discussed below, most samples are uncapped while some are capped with Te, but their properties are not significantly affected due to this discrepancy, especially the ones with high order *n*. All of the films discussed in Figures 3 and 4 have *m* = 10, except for the *n* = 1 sample, which has *m* = 32.

The crystallinity and 2D surfaces of our films are corroborated by reflection high energy electron diffraction (RHEED) images taken *in situ* after growth (Figure S3). Figure 2a shows the X-ray 2θ scans for *n* = 1, 3, 5, 7, and 9 [MnTe]­[Bi_2_Te_3_]_*n*_ films, including peaks belonging to the Al_2_O_3_ substrate and additional peaks due to Te capping in some films. For *n* = 15, i.e., the sample with the largest *n*, due to the extremely long periodicity (>30 nm), the peaks in the XRD pattern are so close to each other that they are included in the Supporting Information, to distinguish the peaks better (Figure S1). Below the XRD scan for each film, the expected values of the (00*l*) reflections based on the *c*-axis lattice constant of each [MnTe]­[Bi_2_Te_3_]_*n*_ are marked as open triangles. The patterns for MnBi_2_Te_4_, and MnBi_6_Te_10_ agree with their counterparts in multiple references.^14,26^ Due to the lack of existence of concrete XRD data for higher order films beyond *n* = 4, their peaks could not be compared with any references, but they match well with their expected values, indicating the formation of ordered structures of [MnTe]­[Bi_2_Te_3_]_*n*_ along the (0001) direction. High-angle annular dark-field scanning transmission electron microscopy (HAADF-STEM) measurements were carried out on one of the samples: MnBi_14_Te_22_. Structurally, MnBi_14_Te_22_ should comprise 6 QLs of Bi_2_Te_3_ inserted between each MnBi_2_Te_4_ SL. These bands of SLs and QLs can be seen in the STEM image in Figure 2b, along with a sharp interface between the film and Te capping, implying a highly ordered growth. The complete STEM image can also be seen in Figure S2. It should be noted here that even though we expect 3 QL of Bi_2_Te_3_ between the Te capping layer and the topmost MnBi_2_Te_4_ septuple layer for the [MnTe]­[Bi_2_Te_3_]_7_ structure based on our growth sequence, the STEM image shows only 2 QL of Bi_2_Te_3_. This implies that Mn/Bi atoms can diffuse vertically over 1–2 nm during the growth. Nonetheless, the overall superlattice periods both in XRD and in STEM are consistent with the designed superlattice order *n*.

**Figure 2:**
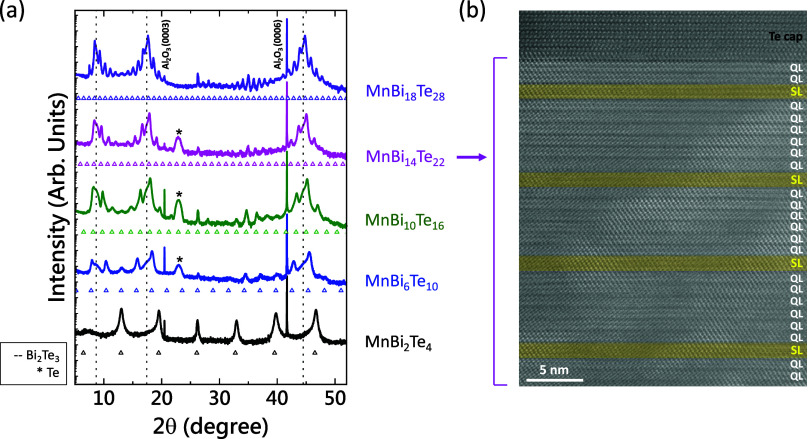
X-ray diffraction patterns and STEM. (a) XRD patterns of [MnTe]­[Bi_2_Te_3_]_*n*_ for *n* = 1, 3, 5, 7, and 9 with their expected peak values represented by open triangles below each pattern. Additionally, Al_2_O_3_ (substrate), Te (capping), and Bi_2_Te_3_ peaks have also been labeled and marked. (b) Cross-sectional HAADF-STEM image of MnBi_14_Te_22_ (*n* = 7) illustrating the MnBi_2_Te_4_ SLs (marked as yellow bands to guide the eye) separated by 6 QLs of Bi_2_Te_3_.

The magnetic transition temperatures for *n* = 1, 2, 3, and 5 as observed from their temperature-dependent sheet resistance have been highlighted in Figure 3a. All samples exhibit metallic behavior. The metallicity also increases with the order of *n*, which is not surprising due to the addition of Bi_2_Te_3_ QLs in each subsequent order. Each of the marked kinks corresponds to the respective magnetic transition temperature. These temperatures are approximately 24, 14, 11, and 9 K for *n* = 1, 2, 3, and 5 respectively, which are in good agreement with their bulk and MBE-grown counterparts.^11–15,27,28^ The type of magnetic transition is better understood from the magnetotransport measurements (*B*∥*c*, 2 K) of these samples. Figures 3b,d,f,h shows the Hall resistance, with the linear part (for *B* > 5 T) subtracted in order to better visualize their magnetic properties. Meanwhile, Figure 3c,e,g,i illustrates the magnetic field dependence of the normalized sheet resistance *R*_*N*_ = (*R* – *R*_*min*_)/(*R*_*max*_ – *R*_*min*_) with *R*_*max*_ and *R*_*min*_ being the maximum and minimum resistance, respectively, allowing us to compare all the samples on the same scale. For *n* = 1 (Figure 3c), an “M shaped” curve is observed with peaks at ±3.5 T, consistent with a spin-flop transition seen in multiple reports.^9,29,30^ The shape of the curve is slightly different from those of bulk crystals, in which there is a plateau instead of a “U shape” between the two spin-flop peaks. However, it is similar to the form observed in other MBE-grown films and gate-controlled exfoliated flakes.^9,30–33^ In Figure 3b, the prominent spike-like features in the Hall resistance coincide with the spin-flop transitions in Figure 3c. We also see a weak hysteretic (FM-like) response in some *n* = 1 films, possibly arising from Mn doping of the Bi_2_Te_3_ QLs.^28,30,34,35^ For *n* = 2, in Figure 3d,e, drastically different properties emerge as the IEC decreases. Instead of a spin-flop, the magnetoresistance curve resembles the signature butterfly shape corresponding to FM order (Figure 3e). We also see two small peaks at ±2.5 T which are likely due to spin-flop of isolated *n* = 1 SLs in the sample.^28,36^ The Hall resistance of *n* = 2 (Figure 3d) yields a hysteresis that looks quite different from that of a typical ferromagnet. This is the characteristic of a spin-flip transition when the interlayer AFM coupling becomes weaker and smaller than the magnetic anisotropy, resulting in a FM-like hysteresis at low temperatures.^16,37^ The coercive field of this hysteresis, *B*_*c*_ = 0.13 T, is consistent with the spin-flip field of bulk MnBi_4_Te_7_.^14–16,27^ Similarly, *B*_*c*_ = 0.08 T for *n* = 3 (Figure 3f) matches well with the spin-flip field of MnBi_6_Te_10_.^12,16,27^ One can see a gradual change of the Hall resistance in the insets of Figures 3d,f,h as *n* increases, with *n* = 5 looking the most like a hysteresis loop typical for FM materials, having *B*_*c*_ = 0.05 T. This is again comparable to previously studied MnBi_10_Te_16_ and MnBi_8_Te_13_,^12,38^ the compounds in which the interlayer AFM coupling ceases to exist.

**Figure 3:**
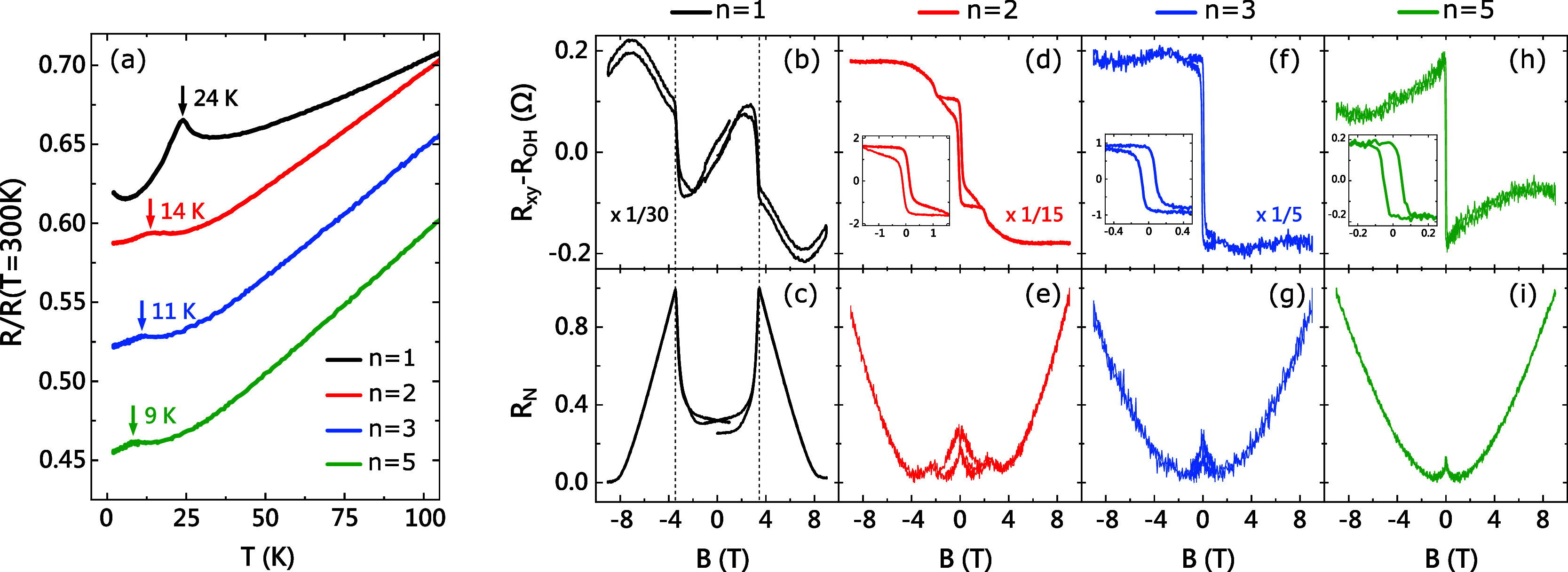
Transport properties of *n* = 1, 2, 3, and 5. (a) Normalized sheet resistance vs temperature plot for *n* = 1, 2, 3, and 5. The magnetic transition temperature for each curve is highlighted. (b, d, f, h) Field dependence of Hall resistance after subtracting ordinary Hall effect in the region *B* > 5 T, for *n* = 1, 2, 3, and 5, respectively. (c, e, g, i) Field dependence of normalized sheet resistance *R*_*N*_ *=* (*R* – *R*_*min*_)/(*R*_*max*_ – *R*_*min*_) for *n* = 1, 2, 3, and 5 respectively. All measurements were taken at 2 K with *B*∥*c*.

Figure 4 presents the temperature and magnetic field dependence of the Hall resistance of the remaining samples, *n* = 7, 9, 11, and 15, that are predicted to be FM: it is notable that this series (*n* ≥ 7) has never been demonstrated before, neither in bulk nor in thin films. The corresponding field dependence of sheet resistance can be seen in Figure S4. When non-FM materials are cooled under a zero magnetic field, the Hall resistance should ideally be zero for all temperatures. But in the case of FM materials, an anomalous Hall signal starts to develop below *T*_*c*_, i.e., when spontaneous magnetization overcomes the thermal fluctuations. Such a signal is observed in the temperature-dependent Hall resistance curves for zero-field-cooled *n* = 7, 9, 11, and 15 samples, which are offset in Figure 4a to easily compare the transition temperatures. Remarkably, clear transitions are seen at around the same temperature, ∼ 10 K, for each *R*_*xy*_(T) curve. Magnetic field sweeps of the Hall resistance of these samples at 2 K yield hysteresis loops, confirming the FM order (Figure 4b). Another common feature among all the samples is that their coercive fields, *B*_*c*_, are all very similar to ∼0.05 T. Both *T*_*c*_ and *B*_*c*_ of *n* = 7, 9, 11, and 15 are similar to those observed for *n* = 5, indicating that all phases for *n* ≥ 5 have the same FM properties. This is possible only if these properties arise solely from the intralayer FM interactions within each SL, and any interlayer coupling is absent (or negligible). On comparing our results with existing literature and theoretical predictions, we conclude that *n* ≥ 4 indeed forms the SLM phase. Such a phase has robust *T*_*c*_ ≈ 10 K and *B*_*c*_ ≈ 0.05 T, completely independent of the number of Bi_2_Te_3_ layers between each magnetic MnTe layer, and is here experimentally proven to survive at least up to *n* = 15.

**Figure 4:**
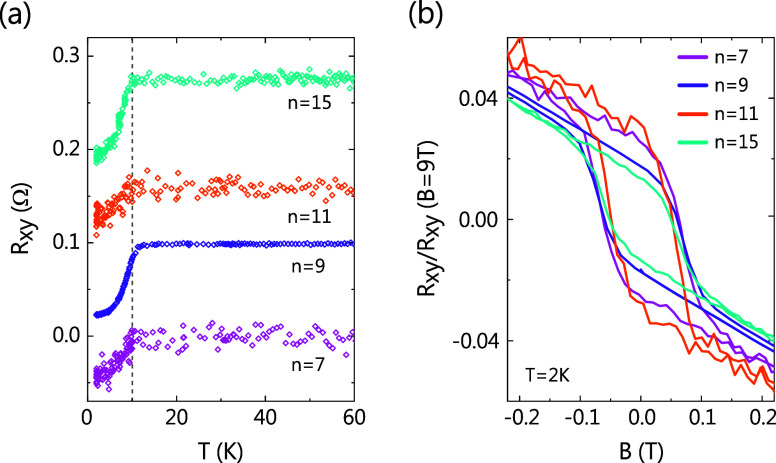
Hall resistance for *n* = 7, 9, 11, and 15. (a) Zero-field-cooled temperature dependence of Hall resistance for *n* = 7, 9, 11, and 15. The curves are offset with respect to each other. (b) Field dependence of normalized Hall resistance for *n* = 7, 9, 11, and 15 at 2 K with *B*∥*c*.

As mentioned before, all of the samples in Figure 4 possess the structure shown in Figure 1b, with each “block” repeated 10 times. Our ability to engineer these structures layer-by-layer allows us to grow them with the desired number of block repetitions. In the following section, we present a systematic study of Hall resistance of *n* = 5 and *n* = 15 samples, the two extremes of the SLM phase in our study, for different number of block repetitions, *m*. In *R*_*xy*_(*T*) of Figure 5c, *T*_*c*_ of ∼10 K is observed for all *m* in *n* = 5, but the corresponding *B*_*c*_ ≈ 0.05 T in Figure 5d is seen only for *m* = 2 and *m* = 3, and *B*_*c*_ becomes almost negligible for *m* = 1. On the other hand, in *n* = 15, *m* = 1 and 2 show no signs of ferromagnetism. Nonetheless, a transition temperature (*T*_*c*_ ≈ 10 K) and hysteresis (*B*_*c*_ ≈ 0.05 T) appear quite abruptly for *m* = 3 and remain almost unchanged until *m* = 10 (Figure 5a,b). For *m* = 1, SLM phase signatures are seen in neither *n* = 5 nor *n* = 15. The FM hysteresis for *m* = 1 in *n* = 5 is very small and could be due to Mn doping of the Bi_2_Te_3_ layers, unrelated to 1 SL ferromagnetism. Similar absence of FM signal has been observed in 1 SL of a MnBi_4_Te_7_ thin flake sample^39^ and has been ascribed to the infeasibility of long-range order in 2D layers as described by the Mermin–Wagner theorem.^40^ The values of *T*_*c*_ and *B*_*c*_ for *m* ≥ 2 in *n* = 5 and *m* ≥ 3 in *n* = 15 are similar to those observed in the samples in Figure 4 and can be attributed to the SLM phase. Interestingly, for *m* = 2, while FM features appear in *n* = 5, there are none in *n* = 15. Ideally, the SLM phase should show up in *m* = 2 for both *n* = 5 and *n* = 15, but its signatures appear at a higher *m* value for *n* = 15 as compared to *n* = 5. This suggests that there is a weak, yet nonzero, long-range interaction between each SL that evidently gets weaker as more Bi_2_Te_3_ QLs are inserted in between. Since the FM features appear after a minimum number of *m* for *n* = 15, we can also infer that this weak interaction is necessary to stabilize the overall FM order. Furthermore, the almost constant values of *T*_*c*_ and *B*_*c*_ imply that this interlayer FM interaction is much weaker than the intralayer superexchange FM coupling. We speculate that this weak interlayer FM coupling could be due to a carrier-mediated RKKY interaction, a dipole interaction between the FM SLs, or both. Multiple studies indicate that defects such as antisite defects and Mn vacancies enhance interlayer FM coupling.^41–45^ Hence, based on our results and the role of such defects in [MnTe]­[Bi_2_Te_3_]_*n*_, artificially introducing a small amount of defects could also help stabilize FM ordering, particularly in high order *n*.

**Figure 5:**
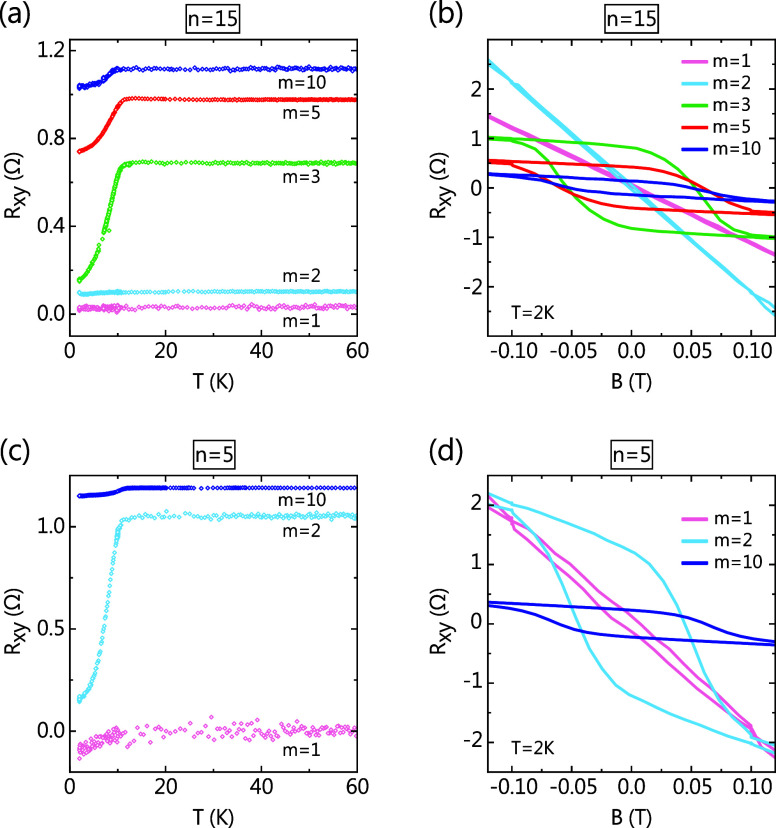
Thickness dependence of the Hall resistance for the SLM phase. Temperature and field dependence at 2 K of (a, b) *n* = 15 and (c, d) *n* = 5 for different values of *m*, where *m* is the number of “block” repetitions in Figure 2.

In summary, by utilizing the ALL-MBE technique, we have synthesized the [MnTe]­[Bi_2_Te_3_]_*n*_ series far beyond that previously reported and revealed their hidden properties in the large *n* limit. The FM signatures for the SLM phase (*n ≥ 5*) are found to be independent of the Mn–Mn distance with *T*_*c*_ and *B*_*c*_ values of ∼10 K and ∼0.05 T, respectively, and survive up to the highest order that we grew, *n* = 15. This observation implies that the FM order is almost entirely determined by the strong intralayer FM interactions within each SL. Nonetheless, the thickness-dependent studies suggest that there should still exist a nonzero interlayer FM coupling between SLs, to stabilize the FM order. Hence, we can conclude that as *n* → ∞, the [MnTe]­[Bi_2_Te_3_]_*n*_ system does not exhibit ferromagnetism. Although the exact origin of this interlayer FM interaction is debatable and requires further in-depth studies, our work demonstrates that there are many untapped natures in the [MnTe]­[Bi_2_Te_3_]_*n*_ family of materials that are hidden beyond the thermodynamic limit.

## Supplementary Material



## Notes

Experimental methods; (Figure S1) XRD pattern of [MnTe]­[Bi_2_Te_3_]_*n*_ for *n* = 15; (Figure S2) STEM image analysis of [MnTe]­[Bi_2_Te_3_]_*n*_ for *n* = 7; (Figure S3) RHEED patterns of [MnTe]­[Bi_2_Te_3_]_*n*_; and (Figure S4) magnetoresistance plots for SLM phase [MnTe]­[Bi_2_Te_3_]_*n*_ (PDF)

## ¶

Materials Science and Technology Division, Los Alamos National Laboratory, Los Alamos, New Mexico 87545, USA

## §

Ningbo Institute of Materials Technology and Engineering, Chinese Academy of Sciences, Ningbo 315201, China.
