# Supplementary material for: Single-Layer Magnet Phase in Intrinsic Magnetic Topological Insulators, [MnTe][Bi2Te3] n , Far beyond the Thermodynamic Limit
Source: Nano Lett. 2025 Mar 13;25(12):4720–6. doi: 10.1021/acs.nanolett.4c05860 (PMC12333432; doi:10.1021/acs.nanolett.4c05860)
Supplement: Supplementary file 1 [file nl4c05860_si_001.pdf]

Supporting Information

Single-layer magnet phase in intrinsic magnetic topological insulators, $[MnTe][Bi_2Te_3]_n$, far beyond the thermodynamic limit

Deepti Jain[1], Hee Taek Yi[1], Xiong Yao[1,6,a], Alessandro R. Mazza[2,3], An-Hsi Chen[2], Kim Kisslinger[4], Myung-Geun Han[5], Matthew Brahlek[2] and Seongshik Oh[1,6]**

[1] Department of Physics and Astronomy, Rutgers, The State University of New Jersey, Piscataway, NJ 08854, USA

[2] Materials Science and Technology Division, Oak Ridge National Laboratory, Oak Ridge, TN 37831, USA

[3] Present address: Materials Science and Technology Division, Los Alamos National Laboratory, Los Alamos, New Mexico 87545, USA

[4] Center for Functional Nanomaterials, Brookhaven National Laboratory, Upton, NY 11973, USA

[5] Condensed Matter Physics and Materials Science, Brookhaven National Laboratory, Upton, NY 11973, USA

[6] Center for Quantum Materials Synthesis, Rutgers, The State University of New Jersey, Piscataway, NJ 08854, USA

[a] Present address: Ningbo Institute of Materials Technology and Engineering, Chinese Academy of Sciences, Ningbo 315201, China

*Corresponding authors' email: jain@physics.rutgers.edu, ohsean@physics.rutgers.edu

Experimental Methods

Thin film growth: All films were grown on $10 \times 10 \times 0.5$ mm^3 Al_2O_3 (0001) substrates using a custom-built MBE system (SVTA) with base pressure of ~10^{-10} Torr. The substrates were cleaned *ex situ* with UV generated ozone followed by *in situ* heating up to 750 °C under oxygen pressure of 1×10^{-6} Torr. The Cr_2O_3 buffer layer was deposited at 700 °C under oxygen pressure of 1×10^{-6} Torr, after which the substrate was cooled down to the growth temperature of $[MnTe][Bi_2Te_3]_n$. The sources used were high purity (99.999%) elemental Bi, Mn, Te, and Cr which were thermally evaporated using standard effusion cells. Source fluxes were calibrated *in situ* with a quartz crystal micro-balance and *ex situ* with Rutherford backscattering spectroscopy.

Transport measurements: The samples were prepared for transport measurement by using manually pressed indium wires in van der Pauw geometry. Magnetoresistance and Hall resistance measurements were carried out in a Physical Property Measurement System (PPMS, Quantum Design inc.) down to 2 K. Keithley 2400 source-measure unit and 7001 switch matrix system, controlled by a LabView program were used to gather data.

XRD and STEM: XRD was carried out, using a Panalytical X'Pert Pro and a monochromated Cu $K_{\alpha 1}$ source. The STEM sample was prepared using a FEI Helios G5 UX focused ion beam system with final Ga+ milling performed at 2 keV. Then, the HAADF-STEM was performed with a JEOL ARM 200CF equipped with a cold field emission gun and spherical aberration correctors, which was operated at 200 kV. The detection angles for HAADF imaging were ranging from 68 to 280 mrad.

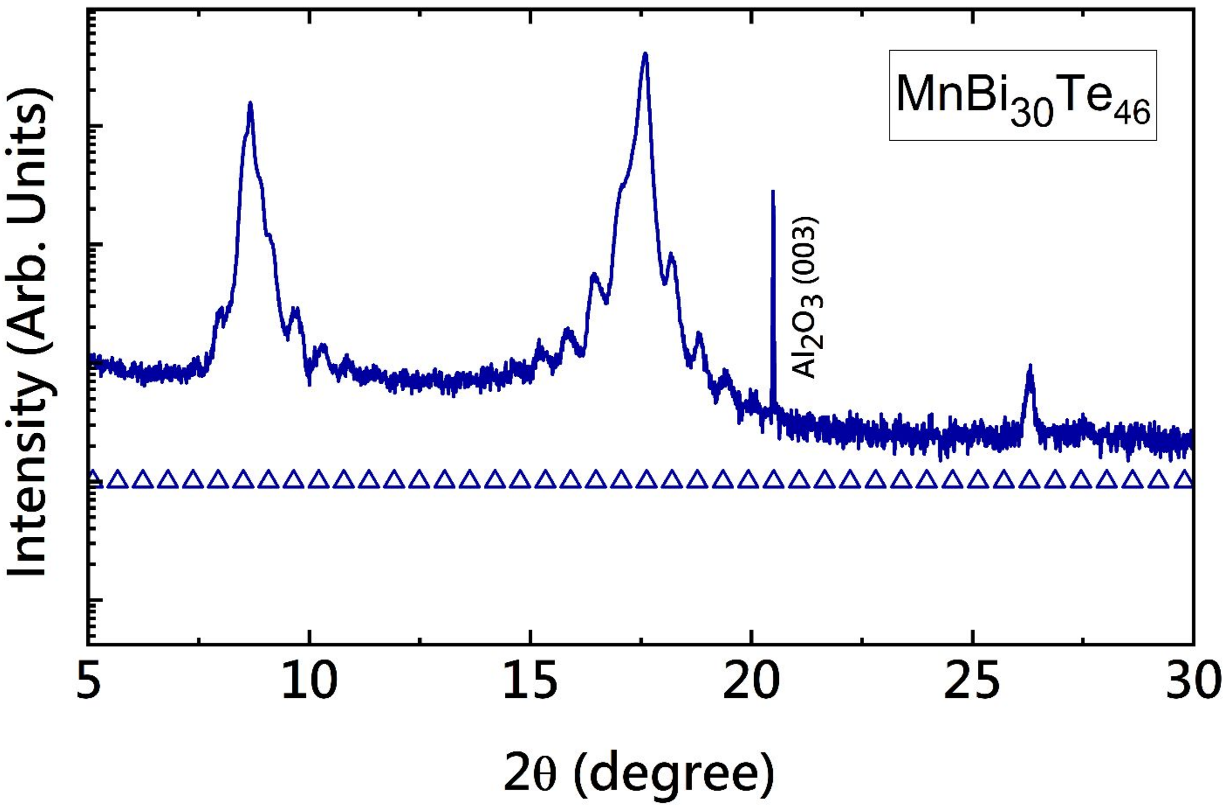

Figure S1 XRD pattern of $[MnTe][Bi_2Te_3]_n$ for n = 15, with the expected peak values represented by open triangles.

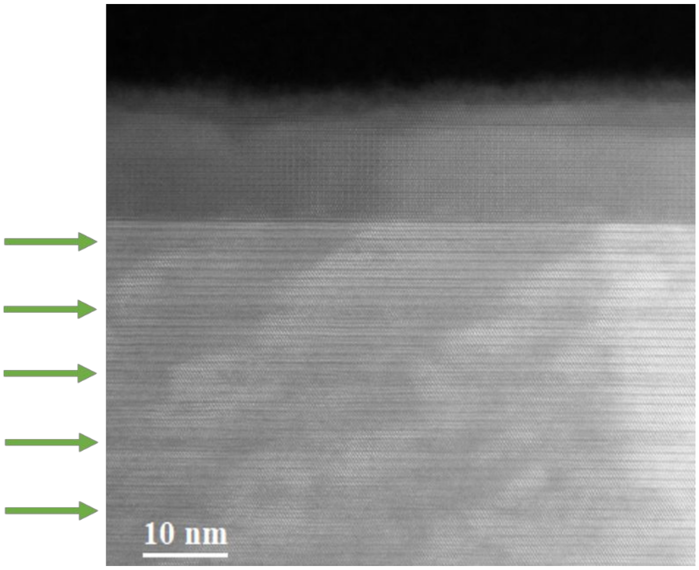

Figure S2 Cross-sectional HAADF-STEM image of $MnBi_{14}Te_{22}$ (n = 7), part of which was shown in Figure 2b. The dark septuple layers have been highlighted with arrows.

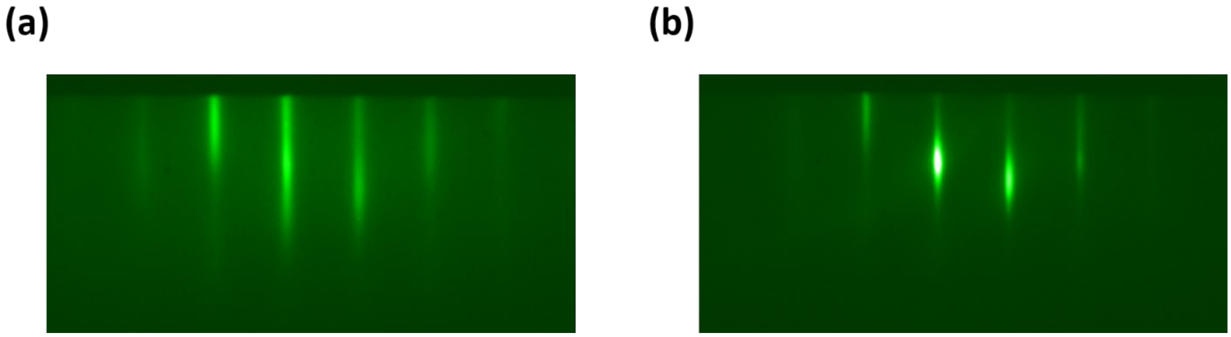

Figure S3 RHEED images of $[MnTe][Bi_2Te_3]_n$ (a) n = 1 and **(b)** n = 5

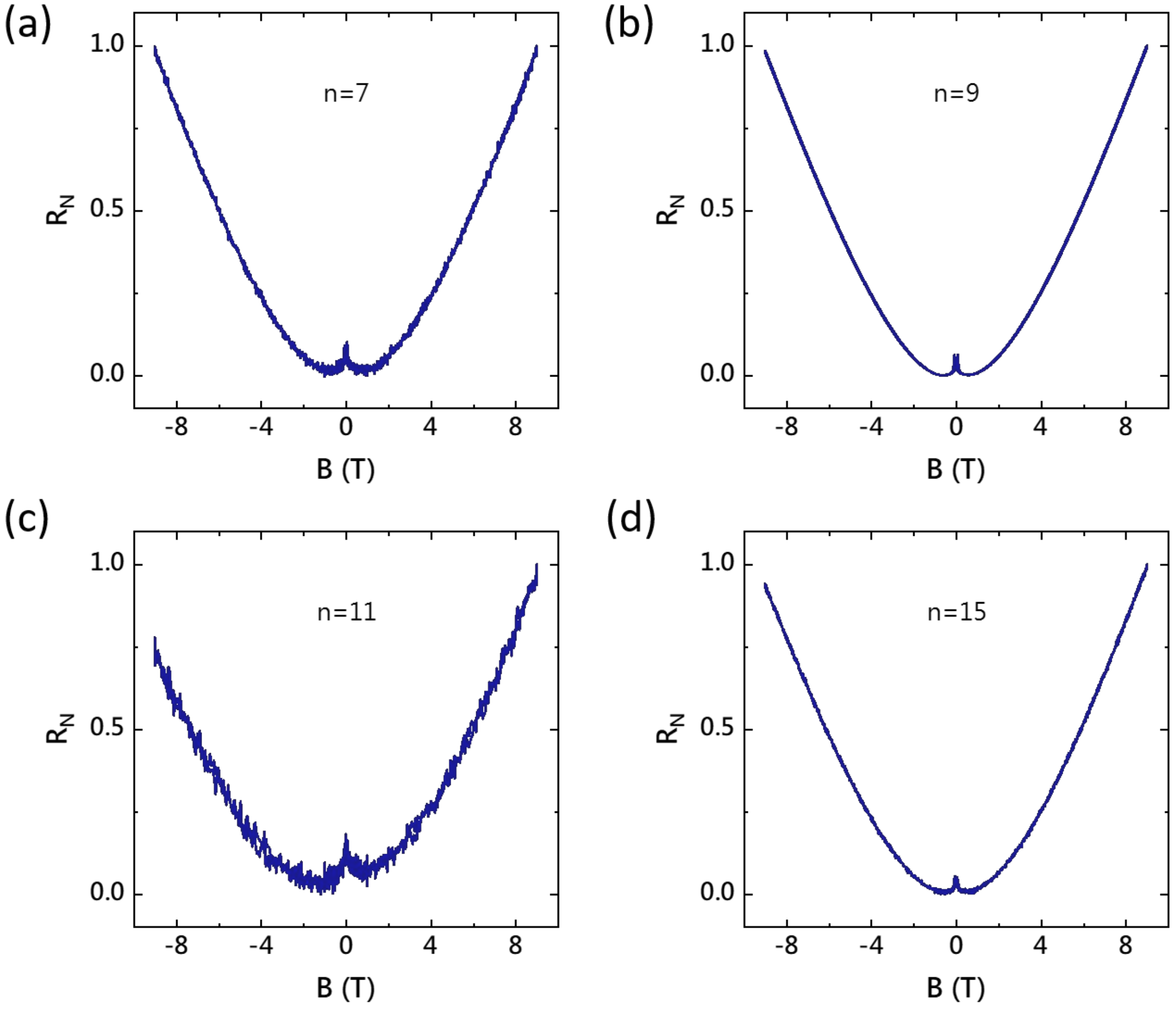

Figure S4 Magnetoresistance plots of *n* = 7, 9, 11 and 15. (a,b,c,d) Normalized sheet resistance $R_N = (R-R_{min})/(R_{max}-R_{min})$ for n = 7, 9, 11 and 15, respectively.
